# Temporal Patterns of Risk Factors for Adjacent Segment Disease After Lumbar Fusion: 5 Years or More and up to 15 Years

**DOI:** 10.3390/jcm14103400

**Published:** 2025-05-13

**Authors:** Jaewan Soh, Hae-Dong Jang, Jae Chul Lee, Taejong Jeong, Byung-Joon Shin

**Affiliations:** 1 Department of Orthopaedic Surgery, Hanyang University Guri Hospital, College of Medicine, Guri 11923, Republic of Korea; md962014@gmail.com; 2 Department of Orthopaedic Surgery, Soonchunhyang University Bucheon Hospital, College of Medicine, Bucheon 14584, Republic of Korea; khaki00@schmc.ac.kr; 3 Department of Orthopaedic Surgery, Soonchunhyang University Hospital, College of Medicine, Seoul 04401, Republic of Korea; jlee@schmc.ac.kr; 4 Department of Orthopaedic Surgery, Sahmyook Seoul Medical Center, Seoul 02500, Republic of Korea; tjjeong86@gmail.com

**Keywords:** adjacent segment disease, early adjacent segment disease, lumbar spinal fusion, risk factors, revision surgery

## Abstract

**Background/Objectives**: There are many concerns regarding adjacent segment disease after lumbar spinal fusion. However, there are few studies that analyze risk factors by classifying adjacent segment disease (ASD) by onset. This study aimed to investigate related factors according to the period of occurrence of ASD in mid- to long-term follow-up patients after lumbar spinal fusion. **Methods**: We analyzed 139 patients who underwent ≤3-segment lumbar fusion for degenerative disease with a minimum 5-year follow-up from a consecutive series of 457 patients. Risk factors for adjacent segment disease (ASD) and early ASD (E-ASD, occurring ≤5 years) were evaluated, including patient factors (age, sex, BMI), preoperative factors (diagnosis, Pfirrmann grade), surgical factors (fusion method, number of segments), and radiological parameters (lumbar lordosis, fused segment angle, PI-LL mismatch). Multivariable Cox proportional hazards modelling and Kaplan–Meier survival analysis were performed to identify independent risk factors. **Results**: A total of 28 patients underwent revision surgery for ASD. Among them, 14 patients developed E-ASD. In the analysis of risk factors for ASD, the fusion method, and the postoperative PI-LL were statistically significant (*p* = 0.003, HR = 4.670, and *p* = 0.008, HR = 3.102, respectively). Regarding E-ASD, the fusion method was statistically significant (*p* = 0.038, HR = 5.444). The cumulative survival rate of ASD was 93.7% at 5 years and 76.4% at 10 years. **Conclusions**: ASD risk factors vary temporally after fusion surgery. Early ASD is primarily associated with the PLIF technique, while long-term risk relates to both the fusion method and sagittal alignment. Surgical planning should consider both the fusion technique and sagittal balance optimization to minimize ASD risk.

## 1. Introduction

The prevalence of degenerative lumbar disease has increased significantly with the ageing population and has extended average life expectancy. Consequently, surgical interventions, particularly instrumental lumbar fusion, have become increasingly common treatment modalities [1,2]. Among various fusion techniques, pedicle screw fixation has emerged as the preferred method due to its superior fusion rates and enhanced biomechanical stability compared to alternative approaches [3]. However, this technique is not without complications; the loss of mobile segments following solid fusion can lead to excessive load distribution and stress concentration on adjacent segments, potentially accelerating their degeneration.

Studies have demonstrated that the incidence of adjacent segment degeneration increases progressively over time following lumbar fusion [4,5,6,7]. Numerous investigations have explored risk factors associated with adjacent segment degeneration during long-term follow-up after fusion surgery. It is important to distinguish between ‘adjacent segment degeneration’, which refers to radiographically evident degenerative changes in adjacent segments, and ‘adjacent segment disease’ (ASD), which describes symptomatic degeneration requiring revision surgery.

While extensive research has examined factors contributing to post-fusion ASD in lumbar degenerative diseases, there are limited studies analyzing the temporal patterns of ASD development and the specific risk factors associated with different time periods during mid- to long-term follow-up. Accordingly, we investigated the temporal occurrence of ASD in patients followed for 5 to 15 years after initial lumbar fusion surgery. This temporal classification enables a detailed analysis of period-specific risk factors and potentially different pathogenic mechanisms. In addition, we examined the survival rate of ASD.

This study aims to enhance our understanding of the natural history of ASD and identify time-dependent risk factors, which could inform surgical decision-making and improve patient long-term outcomes after lumbar fusion surgery.

## 2. Materials and Methods

### 2.1. Patient Population

A total of 457 patients underwent a conventional open lumbar spinal fusion (posterolateral fusion: PLF; posterior lumbar interbody fusion: PLIF) with pedicle screw fixation of three or fewer segments due to degenerative lumbar disease by a single centre from January 2000 to December 2015. PLF is indicated when the endplate is irregular, the disc height is relatively preserved, and there is no foraminal stenosis, whereas PLIF is indicated when the disc height is reduced, foraminal stenosis is present, and the endplate is well maintained. Polyaxial-type pedicle screws were used in all surgeries, and a cage was inserted in the case of PLIF. None of the patients included in the analysis had received a spinal operation prior to the index procedure nor underwent the index procedure under the diagnosis of neoplasm, fracture, dislocation, or infection. Patients who underwent spinal fusion of more than 3 segments or who had a major deformity, such as scoliosis and kyphosis, were also excluded. Cases of pseudarthrosis were also excluded from the study. Among them, 139 patients who could be followed-up for more than 5 years were included in this study. The mean age at initial surgery was 58.5 years (range, 47–75 years), with a mean follow-up duration of 87.7 months (range, 60–180 months).

### 2.2. Factors Considered to Cause ASD

The 139 subjects were investigated retrospectively using their medical records and radiological findings.

### 2.3. Patient-Related Factors

We analyzed demographic factors including sex, age, and body mass index (BMI). Age was dichotomized at 65 years, corresponding to the conventional threshold for elderly classification. BMI was categorized using 25 kg/m^2^ as the threshold for overweight status.

### 2.4. Preoperative Lumbar Spinal Factors

Preoperative diagnoses were categorized into degenerative conditions, such as spinal stenosis, and instability conditions, such as spondylolisthesis. As a preoperative alignment parameter, the lumbar lordotic angle (LLA) from whole-spine standing lateral radiographs was used, measured using Cobb’s method between the upper endplate of L1 and the upper endplate of S1. Adjacent disc degeneration was evaluated using preoperative magnetic resonance imaging (MRI) and classified according to Pfirrmann’s five-grade classification system [8]. Patients with grade ≥ 3 were considered to have significant degeneration. Two spinal surgeons independently assessed disc degeneration grades.

### 2.5. Surgery-Related Factors

Fusion methods were categorized as either PLF or PLIF. The number of fused segments was classified as single-level or multilevel (two or three segments).

### 2.6. Postoperative Radiological Change-Related Factors

Postoperative whole-spine standing lateral radiographs were analyzed for the following measurements:

(1) LLA
(2) Correction of LLA: The difference in correction values between preoperative LLA and postoperative LLA.
(3) Fused segment lordotic angle (FSLA): This is measured between the upper and lower endplates of the fused segments.
(4) FSLA per level: This is calculated as the FSLA divided by the number of fused segments.
(5) Pelvic incidence (PI): This is measured on whole-spine standing lateral radiographs.
(6) PI-LL mismatch: This is calculated as the difference between PI and postoperative LLA, with 10° used as the threshold for analysis.

All radiographic measurements were independently performed by two spinal surgeons, and mean values were used for analysis. Continuous variables were converted to dichotomous variables based on receiver operating characteristic (ROC) analysis to optimize predictive thresholds for ASD.

### 2.7. Criteria for ASD

ASD was defined as the development of symptomatic degenerative changes in adjacent segments requiring revision surgery. Qualifying conditions included spinal stenosis, disc herniation, segmental instability (>3 mm anterior or posterior displacement on sagittal radiographs), or deformity.

### 2.8. Statistical Analysis

Statistical analyses were performed using IBM SPSS Statistics^®^ version 28 (IBM Corp, Armonk, NY, USA). Interobserver reliability was assessed using interclass correlation coefficients for radiographic measurements (LLA, FSLA, PI) and κ statistics for disc degeneration grading. Univariate analysis employed chi-squared tests to evaluate associations between potential risk factors and ASD/E-ASD. Multivariable Cox proportional hazards models were used to identify independent risk factors for both overall ASD and E-ASD, with results expressed as hazard ratios (HRs) with 95% confidence intervals (CIs). The cumulative incidence of ASD requiring revision surgery was analyzed using life table analysis and depicted with Kaplan–Meier survival plots. Statistical significance was set to *p* < 0.05.

### 2.9. Declaration of Generative AI and AI-Assisted Technologies in Writing Process

During the preparation of this work, the authors used [Claude 3.5 Sonnet version/Anthropic] in order to perform grammar proofreading, the refinement of sentences, and validation of statistical analysis. After using this tool, the authors reviewed and edited the content as needed and take full responsibility for the content of the publication.

## 3. Results

### 3.1. ASD

Of the 457 patients who underwent index spinal fusion, 28 (6.1%) required revision surgery for ASD. The anatomical distribution of ASD was predominantly proximal (*n* = 18), followed by distal (*n* = 9) and both proximal and distal segments (*n* = 1). The primary indications for revision surgery were spinal stenosis (*n* = 16), lumbar disc herniation (*n* = 9), and segmental instability/spondylolisthesis (*n* = 3). Surgical interventions included an extension of fusion (*n* = 18), discectomy (*n* = 6), and decompressive laminectomy without fusion (*n* = 4).

Early ASD (E-ASD), occurring within 5 years post-fusion, was observed in 14 patients, with 10 in proximal segments and 4 in distal segments. Treatment consisted of decompression without fusion in five cases and additional fusion surgery in nine cases.

### 3.2. Incidence of ASD

**Figure 1:**
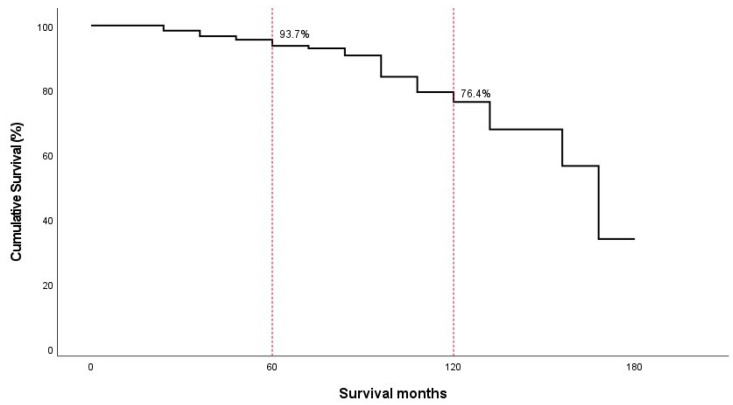
The Kaplan–Meier survival plot shows the predicted cumulative survival of adjacent segments was 89.9% at 5 years and 73.2% at 10 years after surgery.

The annual incidence of ASD requiring revision surgery remained relatively constant at 5.2% (95% CI, 4.4–6.0) over the 15-year follow-up period. Life table analysis revealed cumulative adjacent segment survival rates of 93.7% (95% CI, 92.0–95.4) at 5 years, 76.4% (95% CI, 70.8–81.9) at 10 years, and 33.9% (95% CI, 15.0–52.9) at 15 years (Table 1) (Figure 1).

### 3.3. Analysis of Causative Factors

#### 3.3.1. Patient-Related Factors

In the classification by sex, the incidence of ASD after the initial operation was 21.8% males and 19.0% females, respectively, so there was no statistically significant correlation according to sex (*p* = 0.690). Moreover, depending on age, ASD occurred in 19.4% of those <65 years old and in 26.7% of those ≥65 years old, with no statistically significant difference (*p* = 0.505). Regarding the patients’ BMI, ASD occurred in 18.3% of patients <25 kg/m^2^ and in 22.1% of patients ≥25 kg/m^2^; there were no statistically significant differences (*p* = 0.582).

Similar findings were observed for E-ASD, with no significant correlations for sex (*p* = 0.156), age (*p* = 0.176), or BMI (*p* = 0.516) (Table 2).

#### 3.3.2. Preoperative Lumbar Spinal Factors

Of the patients diagnosed with degenerative disease, 21.6% developed ASD. Furthermore, of patients diagnosed with spinal instability, 18.5% developed ASD. No significant difference was observed in the incidence of ASD between groups (*p* = 0.643).

In the radiographical analysis, the interclass correlation coefficient of the preoperative LLA was 0.981.

In preoperative simple whole-spine standing lateral radiography, the LLA was analyzed based on the value determined by the discriminant thresholds of the ROC analysis of 35° (sensitivity: 88.2%; specificity: 85.8%). ASD occurred in 24.6% of the patients with a postoperative LLA in less than 35° and in 16.2% of the patients with a postoperative LLA in 35° or more; however, the difference was not statistically significant (*p* = 0.218).

The Kappa coefficient of the disc degeneration grading between observers was 0.722, which showed good agreement. On preoperative MRI, according to the Pfirrmann grade, ASD occurred in 18.3% of patients with grade < 3 and 29.2% of patients with grade ≥ 3, and there was no statistically significant difference (*p* = 0.226).

Regarding E-ASD, similar findings were observed, with no significant correlations for preoperative spinal diagnosis (*p* = 0.382), preoperative LLA (*p* = 0.412), or preoperative Pfirrmann grade (*p* = 0.054) (Table 2).

#### 3.3.3. Surgery-Related Factors

According to fusion methods, ASD occurred in 13.7% of patients who underwent PLF and in 23.9% of patients who underwent PLIF, with no statistically significant correlation (*p* = 0.151). Regarding the number of fused segments, ASD occurred in 18.1% of the patients with single-level fusion and 23.2% of the patients with multilevel fusion, but the difference was not statistically significant (*p* = 0.458). In the PLF group, ASD occurred in 1 out of 22 single-level fusion cases and in 6 out of 29 multilevel fusion cases; however, there was no statistically significant correlation (*p* = 0.097). In the PLIF group, ASD occurred in 14 out of 61 single-level fusion cases and in 7 out of 27 multilevel fusion cases; likewise, no statistically significant correlation was found (*p* = 0.763).

Similar findings were observed for E-ASD, with no significant correlations for the fusion method (*p* = 0.067) or number of fused segments (*p* = 0.836) (Table 2).

#### 3.3.4. Postoperative Radiological Change-Related Factors

In the radiographical analysis, the interclass correlation coefficients of the postoperative LLA, FSLA, and PI were 0.984, 0.992, and 0.972, respectively, a result that showed excellent agreement among the two observers.

In postoperative simple whole-spine standing lateral radiography, the LLA was analyzed based on the value determined by the discriminant thresholds of the ROC analysis of 38° (sensitivity: 86.2%; specificity 86.0%). ASD occurred in 23.9% of the patients with a postoperative LLA in less than 38° and in 16.2% of the patients with a postoperative LLA in 38° or more; however, the difference was not statistically significant (*p* = 0.254). In the ROC analysis, the correction of the LLA had a threshold value of 8° (sensitivity: 88.2%; specificity: 89.3%). ASD occurred in 18.4% of cases with less than 8° correction and in 23.1% of cases with 8° or more, but the difference was not statistically significant (*p* = 0.505). Moreover, the postoperative FSLA per level was analyzed based on the value determined by the ROC analysis of 14° (sensitivity 87.5%, specificity 86.4%). ASD occurred in 20.0% of the patients with a postoperative FSLA per level in less than 14° and in 20.3% of the patients with a postoperative FSLA per level in 14° or more, but there was no significant correlation (*p* = 0.966). Based on the angle of 10° for the postoperative PI-LL, ASD occurred in 14.8% of the patients with less than 10° PI-LL, and in 38.7% of the patients with 10° or more PI-LL, there was a statistically significant correlation (*p* = 0.003).

Regarding E-ASD, similar findings were observed, with no significant correlations for the postoperative LLA (*p* = 0.297), the correction of LLA (*p* = 0.305), the postoperative FSLA per level (*p* = 0.977), or the postoperative PI-LL (*p* = 0.051) (Table 2).

#### 3.3.5. Multivariable Cox Proportional Hazards Model

As factors predicted for the occurrence of ASD, sex, age, BMI, preoperative spinal diagnosis, preoperative Pfirmann grade, fusion method of PLF or PLIF, number of fused segments, postoperative LLA, postoperative FSLA per level, and postoperative PI-LL were considered and analyzed by a multivariable Cox proportional hazards model to consider the influence of time variance on all factors.

Regarding ASD, sex (*p* = 0.904), age (*p* = 0.783), BMI (*p* = 0.332), preoperative spinal diagnosis (*p* = 0.448), preoperative LLA (*p* = 0.357), preoperative Pfirrmann grade (*p* = 0.924), number of fused segments (*p* = 0.121), postoperative LLA (*p* = 0.105), correction of LLA (*p* = 0.137), and postoperative FSLA per level (*p* = 0.343) were not statistically significant for the occurrence of ASD. However, when the fusion method was PLIF and the postoperative PI-LL was 10 degrees or higher, statistical significance was achieved (*p* = 0.005, HR = 4.442, 95% CI = 1.576–12.517, and *p* = 0.004, HR = 3.653, 95% CI = 1.502–8.884, respectively) (Table 3).

In addition, the factors affecting E-ASD were also analyzed, including sex (*p* = 0.155), age (*p* = 0.756), BMI (*p* = 0.116), preoperative spinal diagnosis (*p* = 0.419), preoperative LLA (*p* = 0.373), preoperative Pfirrmann grade (*p* = 0.306), number of fused segments (*p* = 0.824), postoperative LLA (*p* = 0.343), correction of LLA (*p* = 0.116), postoperative FSLA per level (*p* = 0.771), and postoperative PI-LL (*p* = 0.052). There was no statistically significant difference for the development of E-ASD. Meanwhile, the fusion method was statistically significant (*p* = 0.038, HR = 5.490, 95% CI = 1.100–27.393) (Table 3).

In the Kaplan–Meier survival analysis, PLIF and 10° or more PI-LL show a significantly lower survival rate than PLF and less than 10° PI-LL for ASD (*p* = 0.038 and *p* = 0.010, respectively).

**Figure 2:**
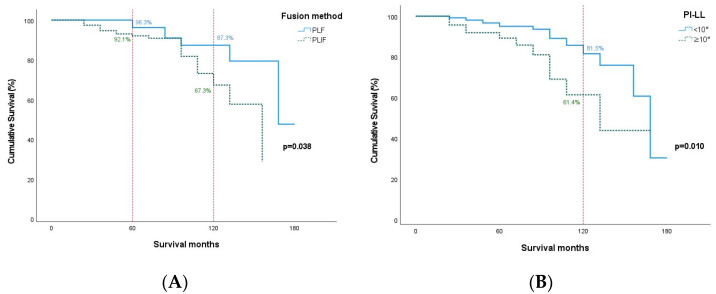
These Kaplan–Meier survival plots show the predicted cumulative survival of adjacent segment disease (ASD) requiring surgery. (**A**) This graph shows the cumulative survival after PLIF was 63.1% at 10 years; however, the cumulative survival after PLF was 87.2% at 10 years (*p* = 0.042). (**B**) This cumulative survival graph shows that 10° or more PI-LL was 55.2% at 10 years but less than 10° PI-LL was 79.5% at 10 years (*p* = 0.006).

The cumulated survival of ASD requiring surgery after PLF was 96.3% (95% CI, 93.7–98.9) at 5 years and 87.3% (95% CI, 81.8–92.9) at 10 years; however, the cumulated survival after PLIF was 92.1% (95% CI, 89.8–94.3) at 5 years and 67.3% (95% CI, 58.4–76.3) at 10 years (Table 4) (Figure 2A). Then, less than 10° PI-LL was 95.0% (95% CI, 93.2–96.8) at 5 years and 81.5% (95% CI, 75.3–87.7) at 10 years but 10° or more PI-LL was 89.2% (95% CI, 84.9–93.5) at 5 years and 61.4% (95% CI, 50.1–72.7) at 10 years (Table 5) (Figure 2B).

## 4. Discussion

The increasing prevalence of degenerative lumbar spinal disease in the ageing population has led to the widespread implementation of instrumented lumbar spinal fusion, with numerous studies reporting favourable outcomes in elderly patients [9,10]. This trend has sparked extensive research into risk factors, surgical techniques, and biomechanical consequences of adjacent segment degeneration following lumbar fusion [6].

The etiology of adjacent segment degeneration remains controversial. Some researchers attribute it to the natural progression of existing degenerative changes or genetic predisposition [9], while others emphasize the biomechanical consequences of fusion, particularly at upper adjacent segments. The latter group has documented increased rates of retrolisthesis, disc herniation, spinal stenosis, and vertebral compression fractures in adjacent segments [11,12]. Multiple factors have been implicated, including rigid fixation, fusion length, pre-existing adjacent segment degeneration, surgical technique, demographic factors, and sagittal balance parameters.

Ha et al. [12] and Kumar et al. [13] reported no gender differences in the occurrence of ASD. However, Etebar and Cahill [14] proposed that adjacent segment degeneration rapidly occurs in postmenopausal women. In this study, we found no gender-related differences in the incidence of ASD and E-ASD.

Our study found no significant gender-related differences in ASD or E-ASD incidence, consistent with findings from Ha et al. [12] and Kumar et al. [13], though contrasting with Etebar and Cahill’s [14] observation of accelerated degeneration in postmenopausal women. Similarly, while previous studies suggested increased ASD risk in older patients [1,4,7,14] and those with higher BMI [15], our analysis revealed no significant associations between age or BMI and ASD/E-ASD development.

A key finding of our study was the significantly higher incidence of both ASD and E-ASD in PLIF compared to PLF patients. PLIF had the benefits of immediate postoperative biomechanical stability and a high fusion rate. The strong mechanical stability of PLIF may possibly increase mechanical stress to adjacent segments and accelerate the postoperative degenerative process of adjacent segments [16]. Lee et al. [17] reported that the incidence of ASD requiring surgery after PLIF was 11.7% at 10 years, but the incidence after PLF was significantly lower, at 6.7% at 10 years, indicating that PLIF was a factor that influenced the occurrence of ASD by 3.4 times. However, Ha et al. [12] and Kim et al. [18] found no differences in the incidence of adjacent segment degeneration according to the instrumental fixation or fusion method, but the changes accelerated when degenerative changes were already present in the adjacent segments before surgery. In this study, we found that the incidence of ASD and E-ASD was higher in patients who underwent PLIF than in those who underwent PLF. In addition, PLIF was the only associated factor with E-ASD occurring within 5 years. Thus, it can be determined that early rigidity can lead to excessive load on the adjacent segment.

In addition, Aota et al. [1] reported that instability worsens after lumbar spinal fusion in patients with spinal instability, such as spondylolisthesis, as a preoperative diagnosis, whereas we found no significant differences in the incidence of ASD and E-ASD according to preoperative diagnosis.

Unlike Aota et al. [1], we found no significant correlation between preoperative diagnosis (instability vs. degenerative conditions) and ASD risk. The debate regarding fusion extent remains active, with some studies suggesting increased ASD risk with multi-level fusion [1,14,19], while others attribute adjacent segment degeneration to sagittal balance rather than mechanical factors [20]. Our findings align with recent studies [10,21], showing no significant association between fusion extent and ASD incidence.

Grouw et al. [22] and Chung et al. [23] proposed that reducing the lordotic angle leads to a concentration of the load and promotes early degenerative changes. Cho et al. [19], Herkowitz and Kurz [24], and Ahn et al. [25] reported that it is important to maintain the LLA after lumbar spinal fusion to achieve good long-term follow-up results. In this study, changes in the lumbar lordotic angle after surgery did not appear to have a significant effect on the occurrence of ASD and E-ASD.

Ahn et al. [25] found that when the postoperative FSLA decreased by 10°, adjacent segment degeneration increased by 3.2 times. Furthermore, if the postoperative FSLA per level is ≥15°, the incidence of adjacent segment degeneration decreases [26]. In this study, however, the postoperative FSLA per level was not associated with the incidence of ASD and E-ASD.

In addition, Rothenfluh et al. [27] reported that if there is a PI-LL mismatch of more than 10 degrees, the incidence of ASD is more than 10 times higher. In this study, there was also a close association with the occurrence of ASD. Although there was no association with E-ASD, in the long-term follow-up, sagittal alignment was found to be associated with the occurrence of ASD. However, given the small number of E-ASD cases (*n* = 14), this may be a result of insufficient statistical power.

Regarding the degeneration of the adjacent disc on preoperative MRI, it did not show significance on the degenerative changes in the adjacent segment in the 5-year follow-up after spinal fusion surgery [26], and in this study, there was no significant effect on the occurrence of ASD and E-ASD.

The incidence of adjacent segment degeneration occurring after lumbar spinal fusion has been reported to be 19.4–40% [1,18]. Aiki et al. [28] performed revision surgery due to adjacent segment disease in 7.7% of patients at a minimum 2-year follow-up, and Gillet [29] reported 78 patients who underwent lumbar posterolateral fusion, with 20% needing revision surgery at more than 5 years of follow-up. In our study, 6.3% required revision surgery for ASD at 5 years and 23.6% required revision surgery for ASD at 10 years.

This study has several limitations that should be considered when interpreting the results. First, the retrospective design limits our ability to establish causal relationships between the observed factors and ASD. Selection bias may have influenced our results, as patients were not randomized to treatment. Second, the number of ASD patients who underwent revision is a relatively small sample size of 28 patients. Because the sample size is relatively small, the generalizability of the study results and the power of statistical analysis may be limited. However, since it is a study of patients with mid- to long-term follow-up of more than 5 years, it is valuable.

## 5. Conclusions

In this longitudinal study of patients undergoing lumbar spinal fusion with 5–15 years of follow-up, we identified distinct temporal patterns in adjacent segment disease (ASD) development and their associated risk factors. The cumulative incidence of ASD requiring revision surgery showed a progressive increase over time, reaching 6.3% at 5 years, 23.6% at 10 years, and 66.7% at 15 years post-fusion.

Our analysis revealed two key findings as follows: first, E-ASD (within 5 years) was significantly associated with PLIF compared to the PLF surgical technique. Second, long-term ASD risk was influenced by both the fusion method and sagittal alignment parameters, particularly PI-LL mismatch.

These findings suggest that surgical planning should carefully consider both the choice of fusion technique and the maintenance of appropriate sagittal alignment to minimize ASD risk. The progressive nature of ASD highlights the importance of long-term surveillance in fusion patients.

## Figures and Tables

**Table 1:** Incidence and survival rate of adjacent segment disease (ASD) requiring revision surgery.

Months	No. of Patients Entered	No. of Patients Withdrawing	No. of Occurrences of ASD	Annual Incidence (%) (95% CI, %)	Cumulated Survival Rate (95% CI, %)
0~12	457	85	0	0	100.0
12~24	372	114	5	1.3 (0.5–2.2)	98.4 (97.7–99.1)
24~36	253	51	4	1.6 (0.5–2.6)	96.7 (95.6–97.8)
36~48	198	36	2	1.0 (0–2.2)	95.6 (94.3–96.9)
48~60	160	18	3	1.9 (0.5–3.2)	93.7 (92.0–95.4)
60~72	139	36	1	0.7 (0–2.1)	92.9 (91.1–94.8)
72~84	102	34	2	2.0 (0.3–3.6)	90.7 (88.4–93.1)
84~96	66	22	4	6.1 (4.0–8.1)	84.1 (80.3–88.0)
96~108	40	9	2	5.0 (2.3–7.7)	79.4 (74.5–84.3)
108~120	29	6	1	3.4 (0.3–6.6)	76.4 (70.8–81.9)
120~132	22	8	2	9.1 (5.5–12.7)	67.9 (60.4–75.4)
132~144	12	4	0	0	67.9 (60.4–75.4)
144~156	8	4	1	12.5 (6.5–18.5)	56.6 (44.5–68.6)
156~168	3	1	1	33.3 (23.6–43.1)	33.9 (15.0–52.9)
168~180	1	1	0	0	33.9 (15.0–52.9)

CI: confidence interval.

**Table 2:** Univariate analysis of demographics-associated factors for adjacent segment disease (ASD).

Risk Factors		Patients	ASD (+) (E-ASD)	ASD (−)	*p*-Value	
					ASD	E-ASD
**Patient-related factors**						
Sex	Male	55	12 (8)	43	0.690	0.156
	Female	84	16 (6)	68		
Age	<65 years	124	24 (11)	100	0.505	0.176
	≥65 years	15	4 (3)	11		
BMI	<25 kg/m^2^	71	13 (6)	58	0.582	0.516
	≥25 kg/m^2^	68	15 (8)	53		
**Preoperative lumbar factors**						
Preoperative spinal diagnosis	Degenerative	74	16 (9)	58	0.643	0.382
	Instability	65	12 (5)	53		
Preoperative LLA	<35°	65	16 (8)	49	0.218	0.412
	≥35°	74	12 (6)	62		
Preoperative Pfirrmann grade	<grade 3	115	21 (9)	94	0.226	0.054
	≥grade 3	24	7 (5)	17		
**Surgery-related factors**						
Fusion method	PLF	51	7 (2)	44	0.151	0.067
	PLIF	88	21 (12)	67		
Number of fused segments	Single level	83	15 (8)	68	0.458	0.836
	2 or 3 levels	56	13 (6)	43		
**Postoperative radiologic changes**						
Postoperative LLA	<38°	71	17 (9)	54	0.254	0.297
	≥38°	68	11 (5)	57		
Correction of LLA	<8°	87	16 (7)	71	0.505	0.305
	≥8°	52	12 (7)	40		
Postoperative FSLA per level	<14°	70	14 (7)	56	0.966	0.977
	≥14°	69	14 (7)	55		
Postoperative PI-LL	<10°	108	16 (8)	92	0.003 *	0.051
	≥10°	31	12 (6)	19		

* *p* < 0.05; E-ASD: early adjacent segment disease; BMI: body mass index; PLF: posterolateral fusion; PLIF: posterior lumbar interbody fusion; LLA: lumbar lordotic angle; FSLA: fused segment lordotic angle; PI-LL: pelvic incidence–lumbar lordosis.

**Table 3:** Multivariable Cox proportional hazards regression analysis for risk of ASD and E-ASD.

	Patients	Hazard Ratio		95% Confidence Interval		*p*-Value	
		ASD	E-ASD	ASD	E-ASD	ASD	E-ASD
**Sex**						0.904	0.155
Male	55			Reference group	Reference group		
Female	84	0.951	0.421	0.421–2.148	0.128–1.387		
**Age**						0.783	0.756
<65 years	124	0.843	0.747	0.249–2.845	0.118–4.716		
≥65 years	15			Reference group	Reference group		
**BMI**						0.332	0.116
<25 kg/m^2^	71			Reference group	Reference group		
≥25 kg/m^2^	68	1.512	2.674	0.656–3.487	0.783–9.126		
**Preoperative spinal diagnosis**						0.448	0.419
Degenerative	74			Reference group	Reference group		
Instability	65	0.722	0.613	0.311–1.674	0.187–2.009		
**Preoperative LLA**						0.357	0.373
<35°	71			Reference group	Reference group		
≥35°	68	0.648	0.552	0.258–1.630	0.150–2.037		
**Preoperative Pfirrmann grade**						0.924	0.306
<grade 3	115			Reference group	Reference group		
≥grade 3	24	0.948	2.073	0.318–2.829	0.513–8.370		
**Fusion method**						* 0.005	* 0.038
PLF	51			Reference group	Reference group		
PLIF	88	* 4.442	* 5.490	1.576–12.517	1.100–27.393		
**Number of fused segments**						0.121	0.824
Single level	83			Reference group	Reference group		
2 or 3 levels	56	2.002	1.154	0.832–4.816	0.325–4.093		
**Postoperative LLA**						0.105	0.343
<38°	71			Reference group	Reference group		
≥38°	68	0.449	0.522	0.170–1.182	0.136–1.998		
**Correction of LLA**						0.137	0.116
<8°	87			Reference group	Reference group		
≥8°	52	1.993	2.663	0.803–4.946	0.786–9.026		
**Postoperative FSLA per level**						0.343	0.771
<14°	70			Reference group	Reference group		
≥14°	69	1.560	1.214	0.623–3.909	0.330–4.456		
**Postoperative PI-LL**						* 0.004	0.052
<10°	108			Reference group	Reference group		
≥10°	31	* 3.653	3.621	1.502–8.884	0.987–13.289		

* *p* < 0.05; ASD: adjacent segment disease; E-ASD: early adjacent segment disease; BMI: body mass index; PLF: posterolateral fusion; PLIF: posterior lumbar interbody fusion; LLA: lumbar lordotic angle; FSLA: fused segment lordotic angle; PI-LL: pelvic incidence–lumbar lordosis.

**Table 4:** Cumulative survival rate of adjacent segment disease (ASD) according to the fusion method.

Group	Months	No. of Patients Entered	No. of Patients Withdrawing	No. of Occurrences of ASP	Cumulated Survival Rate (95% CI, %)
PLF	0~12	178	32	0	100.0
	12~24	146	49	0	100.0
	24~36	97	23	0	100.0
	36~48	74	18	0	100.0
	48~60	56	5	2	96.3 (93.7–98.9)
	60~72	49	8	0	96.3 (93.7–98.9)
	72~84	41	9	2	91.0 (86.6–95.4)
	84~96	30	10	1	87.3 (81.8–92.9)
	96~108	19	5	0	87.3 (81.8–92.9)
	108~120	14	1	0	87.3 (81.8–92.9)
	120~132	13	4	1	79.4 (70.3–88.5)
	132~144	8	3	0	79.4 (70.3–88.5)
	144~156	5	2	0	79.4 (70.3–88.5)
	156~168	3	1	1	47.6 (22.4–72.8)
	168–180	1	1	0	47.6 (22.4–72.8)
PLIF	0~12	279	53	0	100.0
	12~24	226	65	5	97.4 (96.3–98.6)
	24~36	156	28	4	94.7 (92.9–96.4)
	36~48	124	18	2	93.0 (91.0–95.1)
	48~60	104	13	1	92.1 (89.8–94.3)
	60~72	90	28	1	90.9 (88.3–93.4)
	72~84	61	25	0	90.9 (88.3–93.4)
	84~96	36	12	3	81.8 (76.3–87.2)
	96~108	21	4	2	73.2 (65.6–80.7)
	108~120	15	5	1	67.3 (58.4–76.3)
	120~132	9	4	1	57.7 (46.0–69.4)
	132~144	4	1	0	57.7 (46.0–69.4)
	144~156	3	2	1	28.8 (7.6–50.1)

CI: confidence interval; PLF: posterolateral fusion; PLIF: posterior lumbar interbody fusion.

**Table 5:** Cumulative survival rate of adjacent segment disease (ASD) according to PI-LL mismatch.

Group	Months	No. of Patients Entered	No. of Patients Withdrawing	No. of Occurrences of ASP	Cumulated Survival Rate (95% CI, %)
PI-LL	0~12	362	71	0	100.0
<10°	12~24	291	91	2	99.2 (98.6–99.8)
	24~36	198	42	2	98.1 (97.1–99.0)
	36~48	154	29	2	96.7 (95.3–98.0)
	48~60	123	13	2	95.0 (93.2–96.8)
	60~72	108	26	0	95.0 (93.2–96.8)
	72~84	82	30	1	93.6 (91.3–95.8)
	84~96	51	19	2	89.1 (85.3–92.8)
	96~108	30	7	1	85.7 (80.8–90.6)
	108~120	22	3	1	81.5 (75.3–87.7)
	120~132	18	7	1	75.9 (68.0–83.8)
	132~144	10	3	0	75.9 (68.0–83.8)
	144~156	7	4	1	60.7 (45.7–75.7)
	156~168	2	0	1	30.4 (7.6–53.1)
	168–180	1	1	0	30.4 (7.6–53.1)
PI-LL	0~12	95	14	0	100.0
≥10°	12~24	81	23	3	95.7 (93.2–98.1)
	24~36	55	9	2	91.9 (88.4–95.4)
	36~48	44	7	0	91.9 (88.4–95.4)
	48~60	37	5	1	89.2 (84.9–93.5)
	60~72	31	10	1	85.8 (80.5–91.1)
	72~84	20	4	1	81.0 (74.2–87.9)
	84~96	15	3	2	69.0 (59.3–78.8)
	96~108	10	2	1	61.4 (50.1–72.7)
	108~120	7	3	0	61.4 (50.1–72.7)
	120~132	4	1	1	43.8 (27.0–60.7)
	132~144	2	1	0	43.8 (27.0–60.7)
	144~156	1	0	0	43.8 (27.0–60.7)
	156~168	1	1	0	43.8 (27.0–60.7)

CI: confidence interval; PI-LL: pelvic incidence minus lumbar lordosis.

## Data Availability

The data presented in this study are available upon request from the corresponding author.

## Abbreviations

The following abbreviations are used in this manuscript:

ASD	Adjacent segment disease
E-ASD	Early adjacent segment disease
PLF	Posterolateral fusion
PLIF	Posterior lumbar interbody fusion
LLA	Lumbar lordotic angle
FSLA	Fused segment lordotic angle
PI-LL	Pelvic incidence–lumbar lordosis
BMI	Body mass index
